# The future of low-temperature carbon dioxide electrolysis depends on solving one basic problem

**DOI:** 10.1038/s41467-020-19135-8

**Published:** 2020-10-16

**Authors:** Joshua A. Rabinowitz, Matthew W. Kanan

**Affiliations:** Department of Chemistry, Stanford University, 337 Campus Drive, Stanford, CA 94305 USA

**Keywords:** Electrocatalysis, Energy, Electrocatalysis

## Abstract

Carbonate formation is the primary source of energy and carbon losses in low-temperature carbon dioxide electrolysis. Realigning research priorities to address the carbonate problem is essential if this technology is to become a viable option for renewable chemical and fuel production.

## Abstract (web summary)

Low-temperature carbon dioxide electrolysis is an attractive process for sustainable fuel synthesis, but current systems suffer from low efficiency. In this comment, authors discuss the limitations arising from the reaction between carbon dioxide and hydroxide, highlighting the need for new research to address this fundamental problem.

The plummeting cost and daily curtailment of renewable electricity have spurred growing interest in using CO_2_ electrolysis to produce chemicals and fuels. High-temperature solid oxide cells that convert CO_2_ to CO and O_2_ have reached nascent commercialization. Low-temperature CO_2_ electrolysis is an attractive alternative that offers more convenient and flexible operation and the ability to generate multicarbon products such as ethylene, ethanol, and propanol. Over the past 10 years, a dramatic expansion of research in this area has yielded substantial progress in fundamental understanding and prototype devices. Leveraging insights from fuel cells and membrane water electrolyzers, researchers have developed gas diffusion electrode (GDE) cells demonstrating synthetically relevant CO_2_ electrolysis current densities (>100 mA cm^−2^) and promising stability. Despite these advances, the energy efficiency (power-to-product) and carbon efficiency (CO_2_-to-product) of low-temperature CO_2_ electrolysis remain too low to support large-scale applications^1,2^. While much current research is focused on CO_2_ reduction catalyst design, the biggest obstacle to improving performance is an often overlooked basic chemistry problem: the rapid and thermodynamically favorable reaction of CO_2_ with hydroxide (OH^–^) to form carbonate (CO_3_^2–^) imposes steady state electrolysis conditions that result in large voltage and CO_2_ losses. Although recent work has brought attention to some aspects of the CO_3_^2–^ problem^2–4^, it is far more pernicious than what is widely appreciated. Here we explain how CO_3_^2–^ formation compromises efficiency to highlight the need for new research directions that address this problem.

## Hydroxide consumption makes alkaline CO_2_ electrolyzers fuel-wasting devices

**Fig. 1:**
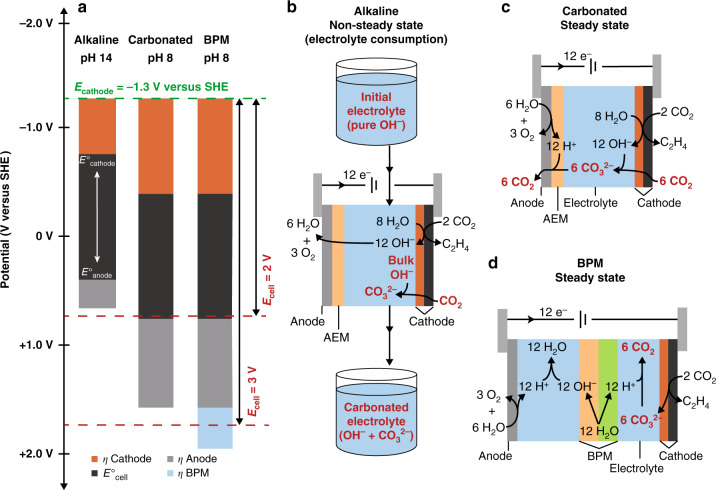
The CO_3_^2–^ problem. **a** Visualization of various contributors to the cell voltage for a CO_2_ electrolysis cell operating under alkaline conditions, carbonated conditions, or with a bipolar membrane (BPM). The contributions shown are the thermodynamic cell potential (*E*°_cell_), and cathode, anode, and BPM overpotentials (*η*). Electrode potentials are referenced to the SHE scale. For simplicity, cell resistance was not included, which would add to the cell voltage. The green dotted line represents the least negative cathode potential reported for low-temperature CO_2_ reduction at >200 mA cm^–2 ^^7^. The red dotted lines provide a visual reference for cell voltages. The thermodynamic potentials for the cathode (*E*°_cathode_) and anode (*E*°_anode_) shift positive versus SHE as the pH is decreased. Alkaline conditions minimize cell voltage but cannot be maintained at steady state because of CO_3_^2–^ formation. **b** Schematic of anion transport in an alkaline flow cell showing OH^–^ consumption by CO_2_. **c** Schematic of a cell at steady state after carbonation showing the carbon loss due to CO_2_ released at the anode. **d** Schematic of a BPM cell at steady state.

The state-of-the-art for low-temperature CO_2_ electrolysis has been obscured by studies that utilize a reservoir of flowing alkaline electrolyte to maintain a high pH in the cell^5–8^. High pH minimizes the cell voltage, which makes these systems appear to have high energy efficiency, but consumption of OH^–^ in the reservoir by CO_3_^2–^ formation results in a net negative energy balance. Understanding why the cell voltage is minimized at high pH helps to clarify the CO_3_^2–^ problem (Fig. 1a). For most known catalyst materials, including Au and Cu, the CO_2_ reduction rate depends on the electron transfer driving force but not explicitly on pH^9–13^. As a result, synthetically relevant current densities require rather negative potentials versus an absolute reference such as the standard hydrogen electrode (SHE). Even with high surface area electrodes, CO_2_ reduction at a geometric current density of >200 mA cm^–2^ has generally required potentials <–1.3 V versus SHE. Because the thermodynamic electrode potentials become more negative versus SHE as the pH is increased, the cathode overpotential is minimized by increasing pH at a fixed cathode potential versus SHE. The overpotential for oxygen evolution at the anode is also generally lowest in base. These contributions significantly reduce the cell voltage at high pH (Fig. 1a).

While minimizing cell voltage is obviously desirable, cells operating under flowing alkaline conditions are not at steady state. The OH^–^ in the electrolyte reservoir is continuously consumed by exergonic CO_3_^2–^ formation as it contacts CO_2_ at the cathode (Eq. 1^14^ and Fig. 1b):

1 $$2{{\mathrm{OH}}^-}_{({\mathrm{aq}})} + {\mathrm{CO}}_{2({\mathrm{g}})} \to {{\mathrm{CO}_3}^{2 - }}_{({\mathrm{aq}})} + {\mathrm{H}}_2{\mathrm{O}}_{({\mathrm{l}})}\,{\Delta}G^\circ = -56\,{\mathrm{kJ}}\,{\mathrm{mol}}^{-1}$$

In practice, the energy required to regenerate CO_2_ and 2 OH^–^ from aqueous CO_3_^2–^ is much larger than |Δ*G*°|, for example >230 kJ mol^–1^ with an optimized system using a calcination cycle^15^. Depending on the product, the energy stored by CO_2_ electrolysis is ~100–130 kJ mol^–1^ of electrons. Thus, the energy balance with flowing alkaline electrolyte will be negative if carbonation consumes ~1 OH^–^ equivalent for each electron of CO_2_ electrolysis current. Though it is rarely quantified, one recent study reported that ~3 OH^–^ were consumed per electron of CO_2_-to-CO electrolysis current with a flowing 1 M KOH electrolyte^8^. Unfortunately, it is common practice to ignore OH^–^ consumption and calculate an “energy efficiency” based solely on the applied voltage, thermodynamic voltage, and faradaic efficiency. These values are qualitatively incorrect. In reality, flowing alkaline CO_2_ electrolysis cells are fuel-wasting devices because it would require more fuel to regenerate the spent electrolyte than the amount of fuel produced by the electrolysis. Using data from flowing alkaline conditions in technoeconomic analyses will grossly overestimate the state-of-the-art for CO_2_ electrolysis.

## Consequences of operating in carbonated electrolyte

Conversion of power to fuel with a positive energy balance requires operating under steady state conditions where there is no net electrolyte consumption. In a cell operating at steady state, CO_3_^2–^ is still formed continuously at the cathode by reaction of CO_2_ with the electrogenerated OH^–^, but it is protonated elsewhere in the cell to release CO_2_. In an anion-transporting cell such as an anion exchange membrane (AEM) cell, CO_3_^2–^ is transported to the anode (Fig. 1c). In order to protonate CO_3_^2–^, the anode pH equilibrates to near-neutral (pH ~ 8)^2^, which increases the cell voltage compared to high pH because the anode thermodynamic potential moves in the positive direction (Fig. 1a). In addition, there is a much greater oxygen evolution overpotential at near-neutral pH with available catalysts. Thus, most of the energy penalty from the CO_3_^2–^ problem is a consequence of what happens at the anode. In fact, the same problem is seen in AEM water electrolysis, where the cathode catalyzes H_2_ evolution. Contamination of an AEM water electrolyzer with trace amounts of air (400 ppm CO_2_) results in the formation of CO_3_^2–^, which causes a >1 V increase to the cell voltage^16^.

The steady state carbon flux imposed by CO_3_^2–^ formation also sets an upper limit on carbon efficiency in anion transporting cells. For CO_2_ reduction to CO, one CO_2_ is released at the anode for every CO that is produced at the cathode, resulting in a maximum carbon efficiency of 50%. For ethylene, the maximum is 25% (Fig. 1c). Many cells operate at much lower carbon efficiencies than these maxima because a large excess of CO_2_ is supplied to the cathode to support high current densities. Low carbon efficiency is a major barrier to large-scale chemical and fuel electrosynthesis because there is a substantial energy and financial cost to obtain a CO_2_ feedstock of suitable purity.

An alternative to an anion transporting cell is to operate with a bipolar membrane (BPM) configured such that CO_2_ electrolysis is coupled with water dissociation at the BPM. At steady state, CO_3_^2–^ generated at the cathode is protonated at the BPM interface, releasing CO_2_ on the cathode side (Fig. 1d). While this design avoids releasing CO_2_ at the anode^17^, it has a lower energy efficiency than an AEM cell because the BPM imposes an additional overpotential in order to drive water dissociation (Fig. 1a). It is important to note that CO_2_ crossover through a BPM is low but non-zero^18^. As such, BPM cells that start with high pH on the anode side will transform to a CO_3_^2–^ electrolyte at steady state.

## Current state-of-the-art and outlook

The consequences of the CO_3_^2–^ problem are evident in the performance that has been demonstrated at steady state. For the production of CO, the best reported performance is for lab-scale (5 cm^2^) devices that have been operated at steady state for >4000 h at ~200 mA cm^–2^ CO_2_-to-CO current density, nicely demonstrating the viability of durable CO electrosynthesis at a reasonable rate. However, the carbon efficiency is 50% and the cell voltage is 3.0 V, corresponding to an energy efficiency of only 43%^1,19^. Both of these values are consistent with the analysis in Fig. 1. To make a more reduced product such as ethylene, the CO_3_^2–^ problem is more pronounced. The best reported performance at steady state is 60 h of operation at ~500 mA cm^–2^ CO_2_-to-ethylene current density, ~2% carbon efficiency, and a cell voltage of 3.9 V, corresponding to an energy efficiency of ~15%^20^. The evaluation of full cell metrics under steady state conditions remains uncommon in CO_2_ electrolysis research, but such experiments are essential for assessing progress and should become standard to demonstrate the impact of a material or design advance.

Low-temperature CO_2_ electrolysis will not be competitive with other electrical energy storage or CO_2_ conversion technologies without major gains in energy and carbon efficiency. Reducing the overpotential of CO_2_ reduction catalysis remains an important objective provided it can be realized under steady state conditions at high current density. Avoiding the losses imposed by CO_3_^2–^ formation demands a much broader research effort that includes strategies to control the formation and clearance of CO_3_^2–^, creative cell designs, and far greater attention to the anode. The progress on the CO_3_^2–^ problem will determine the trajectory of CO_2_ electrolysis in the next 10 years and its impact beyond the laboratory.
